# Phytochemical Profile and Composition of Chickpea (*Cicer arietinum* L.): Varietal Differences and Effect of Germination under Elicited Conditions

**DOI:** 10.3390/plants12173093

**Published:** 2023-08-29

**Authors:** Iza Fernanda Pérez-Ramírez, Diana E. Escobedo-Alvarez, Magdalena Mendoza-Sánchez, Nuria E. Rocha-Guzmán, Rosalía Reynoso-Camacho, Jorge A. Acosta-Gallegos, Minerva Ramos-Gómez

**Affiliations:** 1 Departamento de Investigación y Posgrado de Alimentos, Facultad de Química, Universidad Autónoma de Querétaro, C.U., Cerro de las campanas S/N, Querétaro 76010, Mexico; iza.perez@uaq.mx (I.F.P.-R.);; 2 Unidad de Posgrado, Investigación y Desarrollo Tecnológico (UPIDET), TECNM/Instituto Tecnológico de Durango, Felipe Pescador 1830 Ote., Durango 34080, Mexico; 3 Campo Experimental Bajío (CEBAJ-INIFAP), Carretera Celaya-San Miguel de Allende Km. 6.5, Guanajuato 38010, Mexico

**Keywords:** chickpea (*Cicer arietinum* L.), germination, chemical elicitation, phytochemical profile

## Abstract

Germination is a simple process that improves the nutritional and medicinal values of seeds such as chickpeas. However, the detailed analysis of the phytochemical profile after chemical elicitation during chickpea germination is indispensable when making inferences about its biological properties. Therefore, an evaluation was made of the effect of the chemical inducers salicylic acid (SA, 1 and 2 mM), chitosan (CH, 3.3 and 7 μM), and hydrogen peroxide (H_2_O_2_, 20 and 30 mM) during germination at 25 °C with 70% RH for 4 days on the content of antinutritional and bioactive compounds, including phenolics, sterols, and saponins, in three Mexican chickpea varieties (Blanoro, Patron, and San Antonio) using UPLC-ELSD-ESI-QqQ-MS/MS, UPLC-DAD-ESI-QqQ-MS/MS, and HPLC-DAD-sQ-MS. The highest increase in phenolics and saponins was found in the Blanoro sprouts induced with SA 2 mM, whereas the highest phytosterol content was detected in San Antonio sprouts induced with CH 7 μM. In addition, significant increases in mono-, di-, and oligosaccharides and decreases in antinutritional contents were achieved after germination with most of the elicitation conditions. More importantly, we identified new compounds in chickpea sprouts, such as the lignans matairesinol and secoisolariciresinol, the phenolic compounds epicatechin gallate and methyl gallate, some phytosterols, and the saponin phaseoside 1, which further increased after chemical elicitation.

## 1. Introduction

Chickpea (*Cicer arietinum* L.) is one of the most ancient and consumed legumes around the world; it can be broadly divided into light-yellow-coated (kabuli) and dark-brown-coated (desi) types and grows mainly in areas with temperate and semiarid climates [1,2]. In Mexico, the northwest area is the major production region of white chickpea for exportation, followed by the west-central region [3]. In this regard, the development of light-yellow chickpea grains with superior characteristics has generated new varieties, such as Blanoro, with a grain color and size that is suitable for the international market [4,5]. Furthermore, Blanoro was one of the most promising kabuli types with the highest values of phenolic acids among the selected chickpea genotypes from Mexico [6]. San Antonio is another variety with a pigmented, coated (desi type) seed that is resistant to root rots and has a higher yield than other varieties [7]. In addition, the introduced variety, Patron, is a pigmented seed that originated under genetic improvement in Mexico.

Chickpeas are considered a good source of carbohydrates and proteins of a higher quality than those of other grain legumes, and they are a source of important vitamins and minerals [2,8]; nevertheless, chickpeas contain antinutritional compounds that can impair the utilization of nutrients. These include the protein antinutritional factors, such as trypsin, chymotrypsin, the amylase inhibitors, and lectins, and the non-protein antinutritional factors, such as tannins and phytic acid. However, these compounds can be reduced or eliminated by using processing methods like soaking, cooking or toasting, fermentation, and germination [2,8,9]. In this regard, there is an increased interest in the production of germinated edible seeds for human consumption.

Germination is a simple process that improves the nutritional and medicinal values of seeds and provides edible sprouts that can be consumed as functional foods [10]. This is of major relevance since the determination of the nutritional composition, as well as the bioactive components, of functional foods has a key role in defining daily nutrient intakes at the population level and their association with health effects [11]. In this regard, the phenolic acid profile and many other bioactive compounds that might be considered beneficial as antioxidants are dramatically augmented during germination; in addition, an increase in the isoflavonoid diversity and overall antioxidant capacity of chickpea sprouts (CS) has been reported [10,12,13,14,15,16]. Furthermore, a few other compounds have been identified in chickpea raw seeds, such as lignans, phytosterols, and saponins [2,8,17,18,19], which might also exert health benefits [20,21]. Nevertheless, the effect of germination on the profile of the phytosterols and saponins of germinated chickpeas, as well as after chemical elicitation, has not been reported.

In addition, the synthesis of secondary metabolites can be augmented through the exogenous application of elicitors. In this regard, salicylic acid (SA), chitosan (CH), and hydrogen peroxide (H_2_O_2_) have been proven to enhance seedling growth and to increase the content of total polyphenols and flavonoids in common beans, soybeans, and chickpea seeds during germination by activating the enzymes involved in phenolic compound biosynthesis [17,18,22,23]. However, a well-characterized phytochemical profile, including that of phytosterols and saponins, after chemical elicitation during chickpea germination has not been evaluated.

The evaluation of the influence of agricultural practices, intra-species biodiversity, and environmental factors, among others, might impact the relationships between the food quality and the health benefits [11]. In this regard, the effect of agricultural practices, such as germination, and environmental factors, such as chemical elicitation, on the enhancement of the nutritional and bioactive component composition might also depend on the chickpea variety. Therefore, the aim of this study was to evaluate the effect of chemical elicitors (SA, CH and H_2_O_2_) on three Mexican varieties of chickpea, Blanoro, San Antonio, and Patron, to improve the nutrimental quality and phytochemical profile of the resulting sprouts.

## 2. Results

### 2.1. Increased Percentage of Germination and Radicle Size of Chickpea Seeds after Chemical Elicitation

**Figure 1:**
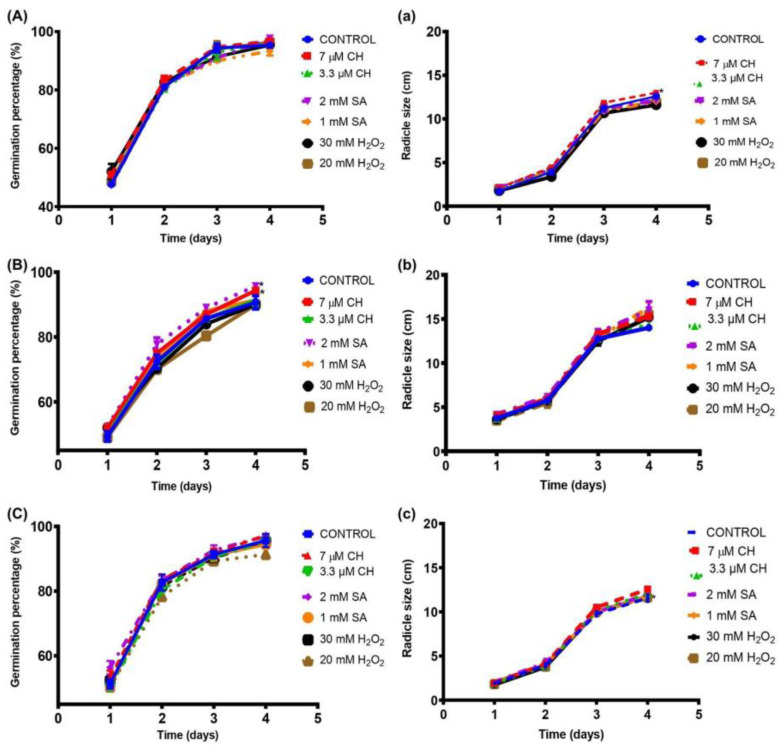
Percentage of germination and radicle size of chemically stressed chickpea sprouts (CS) of three Mexican cultivars. (**A**) Blanoro germination percentage; (**B**) Patron germination percentage; (**C**) San Antonio germination percentage; (**a**) Blanoro CS radicle size; (**b**) Patron CS radicle size; (**c**) San Antonio CS radicle size. Chickpea sprouts (CS) during 4 days of germination at 25 °C with 70% RH in darkness. Data are expressed as mean ± standard deviation of three experimental replicates. * Indicates significant difference (*p* < 0.05) using the Dunnet’s test against control germination. Control: germinated seeds with distilled water. SA: salicylic acid; CH: chitosan; H_2_O_2_: hydrogen peroxide.

The percentage of germination and the radicle size of chickpea seeds treated with three different elicitors were evaluated in order to determine the improvement of the seedling sprouts. The three Mexican chickpea cultivars presented germination percentages higher than 90% after 4 days of germination with or without elicitation; the Blanoro cultivar had the highest increase in germination percentage, particularly the seeds treated with 2 mM SA and 7 μM CH (*p* < 0.05, Figure 1). Similarly, the Patron and San Antonio cultivars elicited with 2 mM SA and 7 μM CH, respectively, showed higher germination percentages compared with their control germination sets, although significant differences were only observed in Patron CS (*p* < 0.1, Figure 1).

According to Figure 1, the 1 mM SA-elicited Blanoro cultivar showed the largest radicle size increase (16.2 vs. 14.0 cm), whereas Patron and San Antonio CS elicited with 7 μM CH had the largest radicle size compared to their control CS. Similarly, with regard to germination percentage, elicitation with H_2_O_2_ showed a negative effect on Patron CS.

### 2.2. Decreased Content of Antinutritional Compounds in Chickpea Sprouts after Chemical Elicitation

Table 1 shows the levels of trypsin inhibitors (TIA), lectins (HU), and phytic acid (PA) in the three raw Mexican chickpea cultivars, and it is clearly observed that the germination process by itself significantly decreases the content of the antinutritional compounds in all CS with respect to their contents in the raw seeds (Table 1). TIA significantly decreased up to 26.2%; the Patron CS had the lowest activity (14.67 ± 0.69 TIU/mg) and the San Antonio CS had the highest decrease (26%) in TIA (from 26.40 ± 1.67 to 19.50 ± 0.12 TIU/mg). Interestingly, all three of the chemical elicitors had an impact on TIA (up to 50.8% inhibition) at the highest applied doses of each elicitor (2 mM SA, 7 μM CH, and 30 mM H_2_O_2_) during germination, as compared to the raw seeds and despite the different initial TIA levels.

Similarly, the content of lectins significantly decreased during germination (up to 51.3%); Blanoro CS had the lowest lectin content. Furthermore, all the chemical elicitors had a significant impact on the lectin content of the CS at the highest concentrations applied (2 mM SA, 7 μM CH, and 30 mM H_2_O_2_), as compared to those of the raw seeds (Table 1). In addition, all three Mexican CS showed the highest decrease in lectin content at 2 mM SA; in particular, the Blanoro sprouts had the highest decrease of 81.2%, as compared to the Patron (73.1%) and San Antonio (61%) CS.

The PA contents in the Blanoro and Patron raw and control CS were similar but were lower for the San Antonio cultivar (Table 1). Despite the differences in the initial content, the PA levels in the CS reached a decrease value of about 40% after 4 days of germination; furthermore, a significant decrease of 69% was observed in Blanoro CS elicited with 1 mM AS and 7 μM CH. In addition, the 30 mM H_2_O_2_-stressed San Antonio sprouts had the largest decrease of 73.1% in PA content (from 4.80 ± 0.46 to 1.29 ± 0.21 mg/g dry flour). Overall, we observed a significant reduction in PA contents after chemical elicitation.

### 2.3. Increased Mono-, Di-, and Oligosaccharide Contents after Germination and Chemical Elicitation

In chickpeas, seed germination exhibits a tight temporal regulation on the release and utilization of sugars (reducing sugars, sucrose, and RFOs). As expected, sucrose occupied first place in terms of abundance in the raw seeds, followed by oligosaccharides and monosaccharides, particularly stachyose and fructose, respectively (Table 2). Interestingly, germination under a control condition (distilled water) significantly increased all the carbohydrate contents in the three Mexican chickpea cultivars; they were further increased or maintained after chemical elicitation. In this regard, the sucrose content was higher in the Blanoro raw seeds; however, the chemically stressed San Antonio and Patron CS reached similar levels of sucrose. Furthermore, San Antonio CS had the highest content of stachyose after chemical elicitation. On the other hand, mannose was only detected in the pigmented San Antonio and Patron raw seeds; however, Blanoro CS had similar mannose contents to that of the pigmented sprouts. It is important to mention that in the present study we identified and quantified mannose, a monosaccharide not previously reported or detected in chickpea seeds and sprouts.

### 2.4. Chemical Elicitation and Chickpea Varietal Effects on Phytochemical Profile of Chickpea Sprouts

Chickpea seeds are a rich source of several polyphenolic compounds. In this regard, the isoflavones biochanin A and formononetin were the major compounds present in the three Mexican CS (Table 3), followed by genistein and daidzein in lower concentrations. The Blanoro CS showed the highest contents of formononetin and biochanin A, followed by the San Antonio and Patron CS, respectively. Regarding the isoflavone genistein, the highest concentration was detected in the control CS of San Antonio, followed by the Blanoro and Patron CS; a similar pattern was observed for the isoflavone daidzein. As expected, the application of chemical elicitors during germination increased the contents of the three major isoflavones (genistein, biochanin A, and formononetin) (Table 3). The greatest increases were detected in the 2 mM SA-stressed Blanoro sprouts (14, 43, and 80%, respectively), the Patron sprouts with 30 mM H_2_O_2_ and 3.3 μM CH treatments (up to 17, 78, and 114%, respectively), and the 7 μM CH-stressed San Antonio sprouts (35, 22, and 45%, respectively).

In the Blanoro CS treated with 2 mM SA, significant increases from 23 to 400% were also detected in the contents of chlorogenic acid, epicatechin, gallic acid, *p*-hydroxybenzoic acid, matairesinol, methyl gallate, ethyl gallate, kaempferol, epicatechin gallate, epigallocatechin gallate, *p*-coumaric acid, rosmarinic acid, quercetin, and sinapic acid; the latter compounds had the highest increases, followed by ethyl gallate and kaempferol. Likewise, the germinated Patron seeds treated with 30 mM H_2_O_2_ and 3.3 μM CH showed the most important increases (30 to 333%) in the contents of chlorogenic acid, epicatechin, daidzein, matairesinol, methyl gallate, ethyl gallate, kaempferol, epigallocatechin gallate, *p*-coumaric acid, rosmarinic acid, quercetin, and rutin, with the highest increase for quercetin, followed by rosmarinic acid, epigallocatechin gallate, and methyl gallate.

Similarly, the germinated San Antonio seeds treated with 7 μM CH showed increases of 21 to 129% in the contents of chlorogenic acid, *p*-hydroxybenzoic acid, matairesinol, ethyl gallate, kaempferol, epicatechin gallate, epigallocatechin gallate, quercetin, and ellagic acid; the latter compounds had the highest increases, followed by ethyl gallate and kaempferol. Interestingly, the flavonoid quercetin was the phenolic compound that commonly showed the highest concentration increases in all three chemically elicited Mexican chickpea cultivars.

As shown in Table 4, β-sitosterol was the major saponin component in the control Mexican CS. Likewise, the compounds campestenyl glucopyranoside and sitosteryl glucopyranoside were identified and quantified in the control Blanoro CS; the compounds campestenyl glucopyranoside, sitosteryl glucopyranoside, and brassicasterol were detected in the control Patron CS, whereas the compounds campesteryl glucopyranoside, fucosterol, and sitosteryl glucopyranoside were also identified in the control San Antonio CS. Although the contents of the phytosterols increased or decreased independently of the elicitor and concentration applied (Table 4), the highest increase in the content of the major component β-sitosterol was detected in all three Mexican CS chemically stressed with 7 μM CH (from 20.5 to 71%), whereas the other phytosterols did not show a clear pattern of induction. Interestingly, we also observed varietal differences in the level of phytosterol induction; the chemically stressed Patron CS had the highest increases (33–3300%) in most of the detected phytosterol compounds, and the San Antonio CS had the lowest increases (8.6–519%) among the three Mexican CS.

Saponins were also detected and quantified in the three Mexican CS, and these included soyasaponins Bb (I), βg (VI), Ba (V), and αg; soyasaponins VI and Af were the major saponins present in the three control Mexican CS (Table 5), followed by soyasaponins Ba (V) and αg; the control Blanoro CS had the highest contents. As expected, the application of chemical elicitors during chickpea germination increased the contents of the major saponins (soyasaponins Af and VI) in all three Mexican CS; the greatest increases were detected with the 2 mM SA treatment for the Blanoro (38 and 25%, respectively); 3.3 μM CH for the Patron (53 and 25%, respectively); and 7 μM CH for the San Antonio cultivars (59 and 35%, respectively). Interestingly, the phaseoside I compound had significantly higher increases in the Blanoro CS (62%), Patron CS (655%), and San Antonio CS (160%) under the same chemical elicitation conditions, respectively. For the rest of the saponins identified in the present study, no significant increases were found in the chemically stressed CS of the three Mexican cultivars.

## 3. Discussion

### 3.1. Increased Percentage of Germination and Radicle Size of Chickpea Seeds after Chemical Elicitation

There is an increased interest in the production of germinated edible seeds for human consumption since germination is a simple process that improves the nutritional value of seeds, obtaining edible sprouts that can be consumed as functional foods [10]. Therefore, it is important to identify the elicitors that stimulate the growth and yield of chickpea sprouts. According to Soltero-Díaz et al. [7], the San Antonio cultivar is a variety with a higher yield than other varieties. In this work, the Patron cultivar showed the highest germination percentages among the three Mexican chickpea cultivars. Interestingly, we observed higher germination percentages in the control seeds of the three Mexican cultivars than those previously reported for other chickpea landraces or varieties [18,22,24]. Although the germination percentage of the three Mexican chickpea seeds in our study was higher (90.0–96.3%) than that previously observed when 5 to 30 mM H_2_O_2_ concentrations were applied (80.0–91.1%) between 2 and 4 d of germination time [23], elicitation with H_2_O_2_ showed the lowest improvement of the seedling sprouts in the three chickpea cultivars, compared to the control seed germination (90.7–95.7%). Similarly, Amjad et al. [17] evaluated the effects of the seed priming technique with H_2_O_2_ on the seed germination and vegetative growth of chickpea seeds and found non-significant improvement compared with the control condition (distilled water). The germination percentage range of the three Mexican chickpea cultivars elicited with SA (91.3–97.7%) was similar to that of chickpeas from Iran germinated with 1.5–3.0 mM SA (90–97%) [22].

On the other hand, the radicle length in the control CS (11.6–14.0 cm) was like those previously reported [24]. In agreement with the results in this work, SA (0.1 and 2 mM) induced a larger radicle size in common bean sprouts; however, no significant difference was observed in sprouted common bean seeds with CH (3.3 and 7 μM) treatment when compared to the control condition (distilled water) [23].

According to the literature, germination growth and radicle size are influenced by environment, including the exposure to chemical substances [17,23,24]. Chemical compounds such as SA, CH, and H_2_O_2_ participate in the regulation of physiological processes such as cell growth, respiration, seed germination, seedling development, and the cell wall formation of radicles [25,26,27]. The evidence points to cell redox status as being the main mechanism associated with plant responses to many abiotic and biotic stresses [26,28] because reactive oxygen species (ROS), particularly H_2_O_2_, participate as signaling molecules involved in the controlling of many different physiological processes, both biotic and abiotic stress responses, throughout the activation of mitogen-activated protein kinase (MAPK) cascades, Ca2+ release, and nitric oxide (NO) synthesis, which are involved in the initiating of environmental stress responses during plant growth and development [26]. In this regard, SA signaling is also connected to cell redox status, and the phenylalanine ammonia lyase (PAL; EC 4.3.1.5) is the key enzyme involved in SA biosynthesis [28]. Similarly, CH induces the accumulation of ROS, such as H_2_O_2_ in the cell wall, upon the wounding of cell tissues. This leads to the induction of plant defense enzymes, including PAL, which is also a main enzyme involved in phenolic compound biosynthesis [27]. In addition, treatment with H_2_O_2_ causes the accumulation of SA [25], thus activating SA signaling within the cell.

### 3.2. Decreased Content of Antinutritional Compounds in Chickpea Sprouts after Chemical Elicitation

The majority of legume plants, including chickpeas, have the ability to synthesize certain biologically active substances which are considered to be antinutritional compounds, causing deleterious consequences to the human digestive system, among other health issues [8,9]. In this regard, the most widely recognized antinutritional compounds from chickpeas are the protease and amylase inhibitors, phytolectins, phytic acid, oligosaccharides, and few other compounds in traces [8,9]. As observed, pigmented or desi type chickpea seeds (Patron and San Antonio) had lower levels of trypsin inhibitors (TIU) than the Blanoro raw (kabuli type) seeds, whereas the mean values for TIU in the seed samples of the desi type were higher compared with the kabuli cultivars grown in India [29]. Even though all three cultivars had higher TIU values than those of the cultivar Blanco Sinaloa 92, also grown in Mexico [18], we can categorize the three cultivars into the low group on the basis of TIU values [29]. Although we did not observe a clear tendency in the hemagglutinin activity (HU) and PA content that depended on the desi or kabuli type, the PA content in all three raw Mexican chickpea cultivars was in the range previously reported [8,29,30,31]. Even though these three cultivars also had a higher PA content than that of the cultivar Blanco Sinaloa 92 [18], we can categorize the three cultivars into the low group on the basis of PA content [29], thus confirming the influence of agricultural practices, wild species, intra-species biodiversity, and environmental factors in the overall composition of chickpeas [11].

According to the literature, sprouting is highly effective in reducing antinutritional compounds from chickpeas and is comparable to the other processing methods [9]. As mentioned before, all three of the chemical elicitors applied had a major inhibition effect on TIU (up to 50.8%, *p* < 0.05) at the highest applied doses of each elicitor (2 mM SA, 7 μM CH, and 30 mM H_2_O_2_) during germination. These results are in accordance with those previously reported by Mendoza-Sánchez et al. [23] in chemically elicited bean seeds. Conversely, optimal germination conditions (30 mM H_2_O_2_ and 72 h germination time) did not significantly reduce the TIU value in the CS of Blanco Sinaloa 92 [18].

Although lectins are present at low levels in chickpeas [32], previous works have observed a decrease in CS from 77% up to the complete elimination of hemagglutinin activity (HU) after 3 and 8 days of germination, respectively [31,32]. However, there are few studies that have investigated the effectiveness of chemical elicitation during germination to reduce lectins in pulses, particularly in CS. According to Mendoza-Sánchez et al. [23], chemical elicitation with SA, CH, and H_2_O_2_ treatments further enhanced lower lectin contents in Dalia common beans, with the highest reduction of 48% in 0.1 mM SA-stressed sprouts as compared to control sprouts (6%) after 3 days of germination at 25 °C in darkness.

Reductions in the PA contents of cereals and legume seeds with sprouting have been frequently reported [1,17,23,27]; this has been attributed to an increase in phytase activities. In this regard, the PA contents in the three Mexican CS were in the range previously reported [26]. In addition, it was found that this antinutrient is more prone to hydrolysis in the case of the kabuli (light-yellow-coated) type than in the desi (dark-coated) type of chickpea [1]. In this work, we found a higher reduction in PA content in the pigmented San Antonio (desi type) than in the non-pigmented Blanoro (kabuli type) CS.

Overall, sprouting reduces the amounts of several antinutrients in the seeds; however, the reduction in PA has been found to be more profound than that of the other compounds [31]. Similarly, a reduction in PA content of up to 40% was higher than those of the TIU values (up to 26%) and HU values (up to 33%) in all three Mexican CS, with the exception of the lectin content in the Blanoro CS (>50%). Furthermore, we observed decreases up to 50.8% and 73% for the TIU values and PA content in the chemically stressed sprouts, respectively, which further supports the fact that chemical elicitation during germination enhances the decrease in antinutritional compounds in legumes [18,19]. In this regard, the results indicated that the 2 mM SA treatment was most effective in reducing the content of lectins and PA, as well as the TIU in the three Mexican CS. These results are in accordance with those previously reported for common beans [18], showing a decrease in the TIA value and PA content by 41.3 and 35.9%, respectively, after germination, which was further enhanced in the 2 mM SA-stressed sprouts by 54.3 and 56.5%, respectively, as compared to the raw bean seeds.

### 3.3. Increased Mono-, Di-, and Oligosaccharide Contents after Germination and Chemical Elicitation

Chickpeas are considered a better source of soluble carbohydrates than other grain legumes, particularly oligosaccharides and sucrose [2,8]. The raffinose family oligosaccharides (RFOs) belong to low-molecular-weight, non-reducing saccharides that in seeds perform very important physiological functions in plant acclimation during seed development, maturation, and germination, among others [33,34]. In this sense, the mono-, di-, and oligosaccharide contents in the three raw Mexican chickpea seeds were in the ranges previously reported for several genotypes and cultivars worldwide [2,31,32,35], but they were higher than the contents of stachyose and raffinose previously reported for fresh Blanoro raw seeds [5].

The evidence on the increases in sucrose after the sprouting of chickpea seeds was reported previously [31,33,35]; the increases are the result of the degradation of RFO catalyzed by the alpha(α)-galactosidase enzyme (EC 3.2.1.22, α-GAL), which provides carbon and energy to the growing seedling [33,36]. In this regard, Arunraj et al. [35] measured α-GAL, RFO, sucrose, and reducing sugars (monosaccharides) during various stages of seed germination in chickpeas and identified the fact that growing tissues immediately start accumulating both sucrose and raffinose after imbibition (24, 48 and 72 h). Similarly, we also observed an increased in the sucrose, RFO, and monosaccharide contents of the three Mexican CS after 4 days of germination, as compared to those values for the raw seed. Similarly, Kalaivani et al. [36] noticed that the accumulation of sucrose and the breakdown of RFOs was regulated to maintain a constant supply of reducing sugars as an energy source during germination. This is of major interest as recent studies have reported that RFO can serve as a prebiotic and can improve the intestine microbial composition in healthy adults; other biological activities, such as anti-allergic, anti-obesity, and anti-diabetic activities and the prevention of non-alcoholic fatty liver disease through the inhibition of lipid accumulation, have also been reported [34,35].

### 3.4. Chemical Elicitation and Chickpea Varietal Effects on Phytochemical Profile of Chickpea Sprouts

According to the literature, sprouting is a highly effective process for increasing bioactive compounds in chickpeas [12,13,14,15,37,38,39]. Therefore, in the present study the phytochemical profile of the germinated seeds was analyzed through an HPLC-MS system, confirming that the isoflavones biochanin A and formononetin [12,16,18,37,38,39] were the major compounds present in the three Mexican CS (Table 3). Although the isoflavone contents of biochanin A, formononetin, and daidzein in the three control Mexican CS were in the range previously reported for chickpeas germinated under similar conditions (≈4 days of germination), it is worth mentioning that the control Blanoro CS showed the highest values compared to those reported in the literature [12,13,38,39]. On the other hand, the genistein content in the three control Mexican CS were higher than those previously reported [38,39], and the San Antonio CS had the highest content.

Additionally, the phenolic compounds previously reported in the chickpea seeds and sprouts were also detected and quantified in the Blanoro, Patron and, San Antonio CS; these included chlorogenic acid, epicatechin, daidzein, the lignans matairesinol and secoisolariciresinol, epigallocatechin gallate, quercetin, and rutin; in lower concentrations, they included catechin, kaempferol, and the protocatechuic, gallic, *p*-hydroxybenzoic, ellagic, *p*-coumaric, and sinapic acids [2,6,8,12,14,15,19,38]. We also identified compounds previously reported in raw Blanoro seeds; these included biochanin A, rutin, catechin, kaempferol, and the gallic, *p*-coumaric, and sinapic acids [6]. Furthermore, it is noteworthy that in this study we identified compounds not previously reported or detected in chickpea sprouts; these included the lignans matairesinol and secoisolariciresinol (>0.10 mg/g), the flavan-3-ol epicatechin gallate, and methyl gallate (<0.10 mg/g), as well as other phenolic compounds in traces (ethyl gallate, rosmarinic acid, and eriocitrin).

The health benefits associated with chickpea consumption are attributed to the two main isoflavones, biochanin A and formononetin, which exert hypolipidemic, anticancer, anti-inflammatory, and antioxidant activities [2,8,13,14,37]. Additionally, the phytoestrogens daidzein and genistein can be extensively metabolized in humans through the activity of the intestinal microbiota to produce metabolites such as equol and enterolactone [2,40]. Similarly, the enterolactone metabolite is formed from the precursors secoisolariciresinol and matairesinol, and these microbial metabolites have increased antioxidant, anti-inflammatory, antineoplastic, and/or apoptotic activities compared to their precursors [40].

As mentioned above, a high increase in the content of the flavonoid quercetin was consistently detected in the chemically stressed Mexican CS (Table 3). This phenolic compound possesses antioxidant and anticancer activities through the induction of the antioxidant enzymes and apoptosis, respectively [41,42]. Likewise, important increases were obtained for the flavonoids kaempferol, epicatechin gallate, and epigallocatechin gallate, which exert anti-viral, anti-bacterial, anti-fungal, anti-inflammatory, antioxidant, anticancer, and cardioprotective properties [41,42,43,44]; epigallocatechin gallate is one of the most widely studied phytochemicals. More recently, the therapeutical anti-obesity and anti-diabetic potential of ethyl gallate was discovered [45], as was the anti-inflammatory and protective effect on intestinal mucosal integrity [44].

Phytosterols previously reported in chickpea oil and seeds were also detected and quantified in the three Mexican CS, and these included β-sitosterol, β-campesterol, avenasterol, stigmasterol, and avenasterol [2,16]. It is important to mention that in the present study we also identified and quantified phytosterol compounds not previously reported or detected in chickpea sprouts, and these included campestenyl glucopyranoside, sitosteryl glucopyranoside, brassicasterol, and fucosterol (>20 μg/g), as well as ergosterol, stigmastanol, and stigmasteryl glucopyranoside (<20 μg/g).

Plant components such as phytosterols, particularly β-sitosterol, exert an anti-tumor effect on multiple malignant tumors, such as those of breast, gastric, lung, kidney, pancreatic, prostate, and other cancers, through molecular mechanisms, such as those which are pro-apoptotic, anti-proliferative, anti-metastatic, and anti-invasive on tumor cells [8,46]. Likewise, the anti-diabetic, antioxidant, anti-inflammatory, and anti-lipidemic activities of phytosterols, such as β-sitosterol, stigmasterol, and their combinations, have also been demonstrated [20,47].

Regarding the content of saponins, the compounds previously reported in the literature in chickpea seeds were also detected and quantified in the CS, and these included soyasaponins Bb (I), βg (VI), Ba (V), and αg [48,49]. Similarly, the germination increased the saponin content in several legumes such as Dalia beans after 3 days of germination [23] and chickpeas after 6 days in the dark [48]. The observed increase in saponin level is most likely due to the activation and synthesis of the various enzymes that enhance the production of secondary metabolites, including the saponins, in response to abiotic stress and are associated with the chickpea cultivar [21]. These results confirm that the three Mexican cultivars evaluated in our study differ in their response to chemical induction in terms of the elicitor and its concentration. In addition, it is important to mention that in the present study we identified and quantified compounds not previously reported or detected in chickpea sprouts, and these included phaseoside 1 and the soyasaponins Bb (I), Af, Bd, and αg.

The health benefits associated with dietary saponins include a wide range of activities, such as anticancer, antimutagenic, hypoglycemic, hypocholesterolemic, hypolipidemic and appetite suppressant, hepatoprotective (against fatty liver formation), immunomodulatory, neuroprotective, anticoagulant, anti-inflammatory, and antioxidant activities, in experimental studies on animals and in in vitro models [21,37,50].

It is important to highlight that the main contribution of our study was the identification of the effect of the germination process; the chemical elicitation in the enhancement of the germination yield, oligosaccharide content, and the concentration and diversity of some phenolic compounds, while diminishing antinutritional compounds, is highly dependent on the chickpea variety. In addition, one of the main findings was the increases detected in the concentration and the diversity of some saponins and phytosterols. Undoubtedly, our study contributes to the field of yield improvement and the possible functional properties of this legume due to the effect of chemical elicitation during germination. Nevertheless, we also consider the fact that our study has several limitations that are related to the sample size analyzed due to limited resources; therefore, it was only possible to focus on specific types of seed or sprouts, with the limitation of having to evaluate the phytochemical profile and some antinutritional factors under one controlled condition of temperature—RH at 4 days of germination, instead of over time; the analytical techniques used, such as UPLC and mass spectrometry, were not readily accessible in all the research laboratories, thus limiting the scope of the investigation; additionally, there was the limitation of scientific evidence on the relationship between the chemical composition of sprouts and their biological activity in in vivo or epidemiological studies through bio-directed phytochemical studies. Further studies are needed to overcome these limitations and to provide more comprehensive insights into the phytochemical composition of sprouts and their health benefits.

## 4. Materials and Methods

### 4.1. Materials

**Figure 2:**
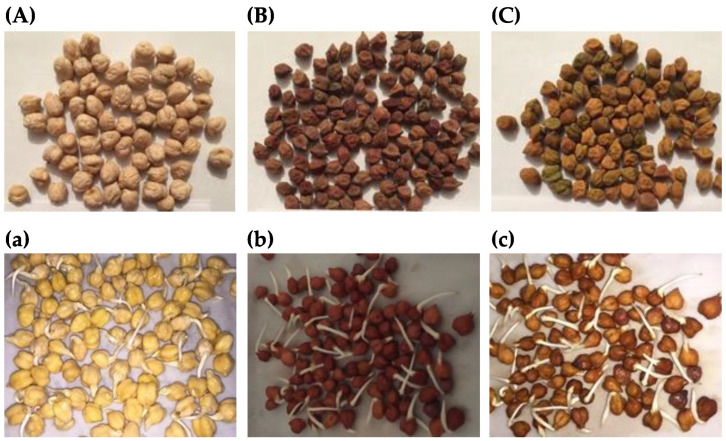
Representative images of raw seeds and sprouts of Mexican chickpea cultivars. (**A**) Blanoro raw seeds; (**B**) Patron raw seeds; (**C**) San Antonio raw seeds; (**a**) Blanoro CS; (**b**) Patron CS; (**c**) San Antonio CS. Chickpea sprouts (CS) after 4 days of germination at 25 °C with 70% RH in darkness.

The chickpea seed (*Cicer arietinum* L.) varieties Blanoro, Patron, and San Antonio were developed and donated by the Instituto Nacional de Investigaciones Forestales, Agrícolas y Pecuarias (INIFAP), Celaya, Guanajuato, Mexico. The chickpea seeds utilized in the trials were obtained during the fall–winter season of 2019–2020 at the Bajío Experimental Station located at 20°34′45.69″ N. 100°49′11.49″ W and 1767 masl. The soil at the site is a Pelic Vertisol with a 1.79% O.M. content. The crop received only the pre-sowing irrigation and a fertilizer rate of 30-30-00 units of N-P-K at sowing. Weeds and insects (leaf miners) were taken care of using the conventional methods. According to the literature, the Blanoro variety is a larger non-pigmented seed which is similar to the kabuli type [5], whereas the Patron and San Antonio cultivars are smaller pigmented seeds like those of the desi type (Figure 2A–C).

### 4.2. Germination Process and Chemical Elicitation Treatments

The seeds (50 g) were disinfected with 1.5% NaClO (1:6 *w*/*v*) for 30 min at room temperature, drained and washed until neutral pH, and then soaked in H_2_O for 24 h. The hydrated seeds were placed in trays between two layers of filter paper moistened with H_2_O or chemical elicitor solutions and were incubated at 25 °C with 70% RH in darkness in a temperature-controlled cabinet for 4 days. Germination was considered when the radicle emerged (Figure 2a–c).

Chemical elicitors were dissolved in distilled water at the following concentrations: salicylic acid (SA, 1 and 2 mM); chitosan (CH, 3.3 and 7 μM); and H_2_O_2_ (20 and 30 mM) [23]. Elicitors were freshly applied every day. Distilled water was used to germinate the seeds of the control group. Triplicate observations were carried out in independent experiments.

The germination percentage was determined based on the total number of seedlings that fully emerged. The lengths of the radicle and plumule (cm) of the germinated seeds were measured and recorded daily. At the end of the experiment, the germinated seeds were immediately plunged into liquid nitrogen, ground in a mill, and passed through a mesh with a particle size of 1 mm; then, the flours were stored at −70 °C to ensure phytochemical stability until further analyses.

### 4.3. Quantitation of Antinutritional Compounds

The determination of trypsin inhibitor activity (TIA) was carried out as reported by Kakade et al. [51]. The TIA was expressed as trypsin inhibitory units (TIU) per mg of dry sample, and one unit of trypsin was defined as the increase of 0.01 absorbance units at 410 nm of reaction mixture. The determination of lectins was carried out using the hemagglutinating activity technique according to the method previously reported by Grant et al. [52]. One unit of hemagglutinating activity (HU) was defined as that contained in the amount of sample in the last dilution which caused 50% agglutination of the blood cells, and the results are expressed as HU/g of dried flour. The phytic acid (PA) content was determined according to the method of Frühbeck et al. [53], and the results are expressed as mg of PA/g of dry sample.

### 4.4. Sample Preparation and Carbohydrate Profile and Mono-, Di-, and Oligosaccharide Contents

The samples (25 mg of dried flours) were extracted with 0.5 mL of 80:20 methanol: water, and sonicated three times for 30 s, with resting of 15 s between cycles. The mixtures were centrifuged at 25,000× *g* for 5 min at 4 °C. The supernatants were recovered, and the extraction procedure was repeated with the residue; then, both supernatants were mixed. After the extraction, the samples were concentrated by using a rotary vacuum evaporator to evaporate the ethanol. Prior to injection, the samples were filtered through a 0.45 μm membrane (Millipore, Bedford, MA, USA).

Monosaccharides (fructose, glucose, and mannose), sucrose, and oligosaccharides (raffinose and stachyose) were identified and quantified by using an ultra-performance liquid chromatograph (UPLC) coupled to an evaporative light-scattering detector (ELSD; Xevo TQ-S, Waters Co., Milford, MA, USA). The samples (2 μL) were injected into a chromatographic column (Acquity BEH Amide, 100 × 2.1 mm, 1.7 μm, Waters Co) using (A) 80:20 (*v*/*v*) acetonitrile:water with 0.1% of ammonium hydroxide and (B) 70:30 (*v*/*v*) acetonitrile:water with 0.1% of ammonium hydroxide as a mobile phase under the following gradient conditions: 5% B at 0 min, 60% B at 5 min, and 60% B at 6 min, followed by a reset and equilibration step for 5.5 min at 250 μL/min. Standard solutions were freshly prepared and injected into the chromatographic system; the resulting peak areas were plotted against concentration for the calibration curve using the external standard method. The results are expressed as μg/g of dry flour [54].

### 4.5. Sample Preparation and Analysis of Polyphenol Profile

The sample preparation and the procedure extraction were performed according to the method reported by a previous study [23]. The polyphenol profile was achieved in an HPLC system with a diode array detector (DAD) coupled to an ESI-sQ MS (Agilent 1200 and 1100 SL).

The samples (10 μL) were analyzed in a Zorbax ODS-C18 (150 × 4.6 mm, 5 μm) column at 30 °C. The mobile phase consisted of (A) 99:1 (*v*/*v*) water:formic acid and acetonitrile (B) at 1 mL/min under gradient conditions. Standard solutions were injected into the chromatographic system; the resulting peak areas were plotted against concentration for the calibration curve. The identification was carried out by comparing their spectroscopic and chromatographic characteristics with those of the standards. Those compounds without commercially available standards were tentatively identified using retention time arrangement, peak spectra, mass-to-charge ratio, MS fragmentation, and online metabolite databases. The results are expressed as μg/g of dry flour [55].

### 4.6. Sample Preparation and Analysis of Phytosterol Profile

The samples (50 mg of dried flours) were extracted with 1 mL of 20:80 dichloromethane/methanol and sonicated three times for 15 s, with resting of 30 s between cycles. Then, the mixtures were centrifuged at 14,000× *g* for 5 min at 4 °C, and the supernatants were evaporated to a concentrate. The samples were dissolved in 1 mL acetonitrile:acetic acid (99.9:0.1) and immediately injected (adapted from Tan et al. [56]).

For the phytosterol separation, the samples were analyzed in a high-performance liquid chromatograph (HPLC) coupled with a DAD and single-quadrupole mass spectrometer (sQ-MS, Agilent 1200). The samples (20 μL) were injected into a Zorbax ODS-C18 (150 × 4.6 mm, 5 μm) column at 35 °C. The mobile phase consisted of methanol (A) and water: acetonitrile 99:1 (B) at 0.8 mL/min under gradient conditions, as previously reported. The measurement of absorbances was performed at 205 nm. The identification was carried out by comparing their spectroscopic and chromatographic characteristics with those of the standards. Those compounds without commercially available standards were tentatively identified using retention time arrangement, peak spectra, mass-to-charge ratio, MS fragmentation, and online metabolite databases [55]. For the quantification of phytosterols, β-sitosterol was used as a standard for the construction of the calibration curve using the external standard method. The results are expressed as µg/g of dry flour.

### 4.7. Sample Preparation and Analysis of Saponin Profile

For saponin extraction, the samples (100 mg of dried flours) were extracted with 1 mL of 80% methanol and stirred for 30 min. Then, the mixtures were centrifuged at 15,000× *g* for 5 min at 4 °C, and the solvent was evaporated. The samples were washed with 1 mL of 70:30 acetone: water, and the solvent was evaporated [23].

The samples (20 μL) were injected into a Zorbax ODS-C18 (150 × 4.6 mm, 5 μm) column at 35 °C. The mobile phase consisted of water containing 8 mM of ammonium acetate (A) and acetonitrile containing 0.1% formic acid (B) at 0.4 mL/min under gradient conditions. The measurement of absorbances was carried out at 310 nm. The identification was carried out by comparing their spectroscopic and chromatographic characteristics with those of the standards. Those compounds without commercially available standards were tentatively identified using retention time arrangement, peak spectra, mass-to-charge ratio, MS fragmentation, and online metabolite databases. For the quantification of the saponins, soyasaponin I was used as standard for the construction of the calibration curve using the external standard method. The results are expressed as µg/g of dry flour [23].

### 4.8. Statistical Analyses

All the results are expressed as mean values ± standard deviation (SD) of triplicate observations. All the variables were parametric; therefore, the data were analyzed by one-way analysis of variance (ANOVA), and differences among the treatments were determined by comparison of the means using the Tukey test and the Dunnet’s test. The statistical significance level was α = 0.05. All the statistical analyses were carried out with JMP 5.0.1 software.

## 5. Conclusions

In conclusion, among the elicitors applied to chickpea seeds, SA induced the highest increase in the percentage of germination, radicle size, and oligosaccharide contents and decreased antinutritional compounds such as lectins, trypsin inhibitors, and phytic acid in all three Mexican chickpea varieties. In addition, SA induced the highest increase in the contents of phenolic compounds and saponins in the non-pigmented Blanoro cultivar. CH elicitation, on the other hand, exerted the greatest effect on the content of phenolic compounds, phytosterols, and saponins of the pigmented Patron and San Antonio varieties, whereas H_2_O_2_ exerted the lowest effect on most of the contents. The diverse effects exerted by the elicitors SA, CH, and H_2_O_2_ on the agronomical parameters, antinutritional compounds, and phytochemical profile suggest that these Mexican chickpea varieties differentially respond to the activation of the signaling pathways that participate in the regulation of physiological processes and of secondary metabolite synthesis. This work confirms that exogenous application of elicitors, such as salicylic acid (SA) and chitosan (CH), on chickpea germination is an effective strategy to improve their nutrimental quality and phytochemical profile with diverse health-promoting activities.

## Figures and Tables

**Table 1:** Content of antinutritional compounds in chemically stressed chickpea sprouts (CS) of three Mexican cultivars.

	Blanoro			Patron			San Antonio		
Treatment	Trypsin Inhibitory Activity ^1^	Haemagglutinating Activity ^2^	Phytic Acid Content ^3^	Trypsin Inhibitory Activity ^1^	Haemagglutinating Activity ^2^	Phytic Acid Content ^3^	Trypsin Inhibitory Activity ^1^	Haemagglutinating Activity ^2^	Phytic Acid Content ^3^
Raw seed	37.8 ± 1.0 a	7.0 ± 0.2 a	0.61 ± 0.04 a	18.7 ± 0.6 a	7.8 ± 0.3 a	0.62 ± 0.03 a	26.4 ± 1.7 a	6.4 ± 0.5 a	0.48 ± 0.05 a
Control ^4^	28.9 ± 0.9 b	3.4 ± 0.4 b	0.37 ± 0.03 b	14.7 ± 1.2 b	5.4 ± 0.5 b	0.37 ± 0.02 b	19.5 ± 1.6 b	4.3 ± 0.2 b	0.29 ± 0.03 b
1 mM SA	19.5 ± 0.8 e	2.4 ± 0.3 d	0.19 ± 0.01 d	10.9 ± 0.8 cd	4.1 ± 0.2 c	0.34 ± 0.01 bc	15.9 ± 0.8 de	3.2 ± 0.3 cd	0.22 ± 0.01 bc
2 mM SA	19.3 ± 1.5 e	1.3 ± 0.1 e	0.29 ± 0.02 c	9.2 ± 0.8 e	2.1 ± 0.1 e	0.30 ± 0.02 cde	13.1 ± 0.1 f	2.5 ± 0.3 e	0.17 ± 0.02 cd
3.3 μM CH	26.5 ± 2.3 bc	2.8 ± 0.1 c	0.22 ± 0.0 b c	12.1 ± 0.2 c	3.3 ± 0.3 d	0.31 ± 0.02 cd	18.9 ± 1.0 bc	3.4 ± 0.2 c	0.23 ± 0.03 bc
7 μM CH	23.9 ± 2.1 cd	1.4 ± 0.1 e	0.19 ± 0.01 d	10.7 ± 0.3 d	3.0 ± 0.1 d	0.25 ± 0.02 e	14.8 ± 1.0 ef	3.0 ± 0 cde	0.20 ± 0.01 cd
20 mM H_2_O_2_	24.6 ± 0.7 cd	3.0 ± 0.1 c	0.29 ± 0.02 c	14.7 ± 0.4 b	3.9 ± 0.2 c	0.30 ± 0.02 cde	18.1 ± 0.5 bc	3.4 ± 0.4 c	0.20 ± 0.02 cd
30 mM H_2_O_2_	22.5 ± 0.9 d	2.1 ± 0.2 d	0.34 ± 0.03 bc	14.0 ± 0.6 b	3.0 ± 0.1 d	0.28 ± 0.02 de	16.9 ± 0.8 cd	2.8 ± 0.2 de	0.13 ± 0.02 d

Data are presented as mean ± standard deviation of three experimental replicates. Different letters in the same column indicate significant (*p* < 0.05) difference by Tukey test. ^1^ Trypsin inhibitory units (TIU)/mg of dried flour, ^2^ Haemagglutinating units (HU)/g of dried flour, ^3^ mg of phytic acid (PA)/100 g of dried flour. ^4^ Control: germinated seeds with distilled water. SA: salicylic acid, CH: chitosan, H_2_O_2_: hydrogen peroxide. Samples were analyzed on fourth day of germination.

**Table 2:** Carbohydrate profile of chemically stressed chickpea sprouts (CS) of three Mexican cultivars.

	Monosaccharides			Disaccharides	Oligosaccharides	
	Fructose	Glucose	Mannose	Sucrose	Raffinose	Stachyose
Retention time (min)	4.39	4.92	4.77	5.69	6.84	7.68
*m*/*z*	179	179	179	341	503	665
Fragments	71, 89	89, 119	89, 119	89, 119, 179	89, 179, 323	89, 179, 323
Treatment	Blanoro (g/100 g)					
Raw seed	0.19 ± 0.01 e	0.03 ± 0.00 d	LDL	7.01 ± 0.02 d	0.10 ± 0.10 d	1.20 ± 0.01 d
Control	0.70 ± 0.01 c	0.45 ± 0.01 a	0.53 ± 0.01 d	12.50 ± 0.02 b	0.72 ± 0.01 b	1.46 ± 0.01 c
2 mM SA	0.82 ± 0.01 a	0.40 ± 0.01 b	0.77 ± 0.02 b	13.88 ± 0.16 a	0.76 ± 0.01 a	1.59 ± 0.04 b
7 μM CH	0.62 ± 0.00 d	0.32 ± 0.02 c	0.96 ± 0.02 a	11.92 ± 0.07 c	0.66 ± 0.01 c	1.63 ± 0.04 b
30 mM H_2_O_2_	0.77 ± 0.01 b	0.43 ± 0.01 ab	0.72 ± 0.01 c	13.96 ± 0.01 a	0.68 ± 0.00 c	1.75 ± 0.01 a
Treatment	Patron (g/100 g)					
Raw seed	0.27 ± 0.00 e	0.01 ± 0.00 e	0.18 ± 0.02 e	3.31 ± 0.02 d	0.15 ± 0.00 d	0.92 ± 0.02 b
Control	0.76 ± 0.02 a	0.47 ± 0.01 a	0.31 ± 0.00 d	12.43 ± 0.10 a	0.38 ± 0.00 c	0.46 ± 0.01 d
2 mM SA	0.47 ± 0.02 c	0.40 ± 0.01 b	0.97 ± 0.01 b	11.54 ± 0.11 b	0.37 ± 0.00 c	0.44 ± 0.00 d
7 μM CH	0.55 ± 0.02 b	0.28 ± 0.01 c	0.93 ± 0.01 c	11.37 ± 0.07 b	0.49 ± 0.01 b	0.78 ± 0.01 c
30 mM H_2_O_2_	0.40 ± 0.01 d	0.12 ± 0.01 d	1.35 ± 0.03 a	10.56 ± 0.01 c	0.64 ± 0.00 a	1.58 ± 0.01 a
Treatment	San Antonio (g/100 g)					
Raw seed	0.11 ± 0.00 c	0.01 ± 0.00 b	0.09 ± 0.01 d	2.60 ± 0.01 c	0.14 ± 0.01 d	0.99 ± 0.02 b
Control	0.48 ± 0.01 b	0.28 ± 0.01 a	0.73 ± 0.03 b	11.48 ± 0.12 b	0.30 ± 0.00 c	0.32 ± 0.00 c
2 mM SA	0.65 ± 0.02 a	0.29 ± 0.01 a	0.72 ± 0.02 b	12.14 ± 0.08 a	0.78 ± 0.00 ab	2.15 ± 0.01 a
7 μM CH	0.49 ± 0.02 b	0.27 ± 0.02 a	0.67 ± 0.02 c	11.58 ± 0.08 b	0.74 ± 0.03 b	2.17 ± 0.03 a
30 mM H_2_O_2_	0.65 ± 0.00 a	0.30 ± 0.01 a	0.92 ± 0.04 a	12.63 ± 0.06 a	0.80 ± 0.01 a	2.17 ± 0.02 a

Data are presented as mean ± standard deviation of three experimental replicates. Different letters in the same column within chickpea variety indicate significant difference (*p* < 0.05) by Tukey test. Control: germinated seeds with distilled water. SA: salicylic acid, CH: chitosan, H_2_O_2_: hydrogen peroxide. Samples were analyzed on fourth day of germination.

**Table 3:** Polyphenol profile of chemically stressed chickpea sprouts (CS) of three Mexican cultivars.

Phenolic Compounds	Retention Time	*m*/*z*	Control	1 mM SA	2 mM SA	20 mM H_2_O_2_	30 mM H_2_O_2_	3.3 μM CH	7 μM CH
			Blanoro						
Chlorogenic acid	1.9	353.1, 191.1 179.0	0.26 ± 0.01 b	0.26 ± 0.00 b	0.32 ± 0.02 a	0.20 ± 0.00 d	0.23 ± 0.00 c	0.25 ± 0.00 b	0.23 ± 0.00 c
Epicatechin	2.0	289.1, 203.1 109.1	0.32 ± 0.01 b	0.30 ± 0.00 b	0.43 ± 0.00 a	0.24 ± 0.01 d	0.28 ± 0.00 c	0.30 ± 0.00 b	0.27 ± 0.00 c
Catechin	3.0	289.1, 203.1 109.1	0.05 ± 0.00 b	0.05 ± 0.00 b	0.06 ± 0.00 a	0.05 ± 0.00 b	0.06 ± 0.00 a	0.05 ± 0.00 b	0.06 ± 0.00 a
Gallic acid	3.7	169.0, 125.0	0.02 ± 0.00 b	0.02 ± 0.00 b	0.03 ± 0.00 a	0.02 ± 0.00 b	0.02 ± 0.00 b	0.02 ± 0.00 b	0.02 ± 0.00 b
*p*-hydroxybenzoic acid	4.2	137.0, 108.0	0.01 ± 0.00 b	0.02 ± 0.00 a	0.02 ± 0.00 a	0.01 ± 0.00 b	0.01 ± 0.00 b	0.02 ± 0.00 a	0.02 ± 0.00 a
Genistein	5.2	269.0, 133.0	1.60 ± 0.01 b	1.47 ± 0.01 d	1.83 ± 0.01 a	1.28 ± 0.01 f	1.28 ± 0.00 f	1.56 ± 0.01 c	1.33 ± 0.00 e
Daidzein	5.6	255.1, 91.0	0.29 ± 0.01 bc	0.31 ± 0.00 b	0.34 ± 0.00 a	0.23 ± 0.00 f	0.24 ± 0.00 ef	0.28 ± 0.00 c	0.25 ± 0.00 de
Matairesinol	6.2	357.1, 122.0 83.1	0.29 ± 0.00 b	0.29 ± 0.00 b	0.37 ± 0.00 a	0.20 ± 0.00 e	0.23 ± 0.00 d	0.27 ± 0.00 c	0.26 ± 0.00 c
Methyl gallate	7.1	167.0, 140.0 124.0, 111.1	0.07 ± 0.00 bc	0.08 ± 0.00 b	0.11 ± 0.00 a	0.06 ± 0.00 cd	0.05 ± 0.00 d	0.06 ± 0.00 cd	0.08 ± 0.00 b
Secoisolariciresinol	9.6	361.2, 346.1 165.0, 121.1	0.10 ± 0.00 b	0.10 ± 0.00 b	0.10 ± 0.00 b	0.10 ± 0.00 b	0.10 ± 0.00 b	0.32 ± 0.00 a	0.10 ± 0.00 b
Ethyl gallate	12.1	197.0, 169.0 124.0	0.01 ± 0.00 b	0.01 ± 0.00 b	0.04 ± 0.00 a	0.01 ± 0.00 b	0.01 ± 0.00 b	0.01 ± 0.00 b	0.01 ± 0.00 b
Kaempferol	14.1	285.1, 151.0	0.05 ± 0.00 b	0.06 ± 0.00 a	0.10 ± 0.00 c	0.05 ± 0.00 b	0.05 ± 0.00 b	0.05 ± 0.00 b	0.05 ± 0.00 b
Protocatechuic acid	14.5	315.1, 123.0 108.0	0.02 ± 0.00 a	0.02 ± 0.00 a	0.02 ± 0.00 a	0.01 ± 0.00 b	0.01 ± 0.00 b	0.02 ± 0.00 a	0.02 ± 0.00 a
Epicatechin gallate	14.7	441.1, 331.1 289.1, 168.9	0.05 ± 0.00 b	0.05 ± 0.00 b	0.08 ± 0.00 a	0.04 ± 0.00 c	0.04 ± 0.00 c	0.05 ± 0.00 b	0.05 ± 0.00 b
Epigallocatechin gallate	16.0	457.1, 331.1 287.1, 169.1	0.20 ± 0.00 e	0.28 ± 0.00 c	0.33 ± 0.00 b	0.19 ± 0.00 e	0.19 ± 0.00 e	0.37 ± 0.00 a	0.25 ± 0.00 d
*p*-Coumaric acid	16.2	163.0, 146.0 119.1	0.09 ± 0.00 cd	0.11 ± 0.00 b	0.15 ± 0.00 a	0.08 ± 0.00 d	0.08 ± 0.00 d	0.10 ± 0.00 bc	0.09 ± 0.00 cd
Rosmarinic acid	16.7	359.1, 197.1 179.1	LDL	LDL	0.002 ± 0.000	LDL	LDL	LDL	LDL
Quercetin	17.2	301.1, 179.1 151.1	0.19 ± 0.00 ef	0.27 ± 0.01 b	0.55 ± 0.00 a	0.18 ± 0.00 f	0.20 ± 0.00 de	0.24 ± 0.00 c	0.24 ± 0.00 c
Formononetin	17.4	267.1, 252.1 191.1	3.37 ± 0.03 e	4.59 ± 0.06 b	6.08 ± 0.06 a	3.22 ± 0.01 f	3.24 ± 0.01 f	4.05 ± 0.01 c	4.10 ± 0.02 d
Biochanin A	18.9	283.1, 268.1 211.1, 109.1	4.46 ± 0.03 d	5.44 ± 0.07 c	6.37 ± 0.05 a	4.37 ± 0.01 d	4.45 ± 0.04 d	5.66 ± 0.01 b	5.33 ± 0.09 c
Sinapic acid	21.3	223.1, 208.0	0.001 ± 0.000 c	0.002 ± 0.000 b	0.005 ± 0.000 a	LDL	LDL	LDL	LDL
Eriocitrin	21.5	595.2, 287.1 151.0	LDL	LDL	LDL	LDL	LDL	0.003 ± 0.00 b	0.01 ± 0.00 a
Ellagic acid	22.5	301.0, 229.0 185.0, 145.0	0.003 ± 0.000 b	0.002 ± 0.000 c	0.01 ± 0.00 a	0.003 ± 0.000 b	0.002 ± 0.000 c	0.01 ± 0.00 a	0.01 ± 0.00 a
Rutin	27.0	609.1, 301.1 151.1	0.21 ± 0.00 b	0.21 ± 0.00 b	0.22 ± 0.00 b	0.27 ± 0.00 a	0.28 ± 0.00 a	0.28 ± 0.00 a	0.28 ± 0.00 a
			Patron						
Chlorogenic acid	1.9	353.1, 191.1 179.0	0.32 ± 0.00 d	0.27 ± 0.00 e	0.38 ± 0.01 b	0.35 ± 0.00 c	0.40 ± 0.00 a	0.42 ± 0.00 a	0.38 ± 0.00 b
Epicatechin	2.0	289.1, 245.1 123.1	0.43 ± 0.00 f	0.39 ± 0.00 g	0.60 ± 0.00 c	0.49 ± 0.00 e	0.70 ± 0.01 a	0.66 ± 0.00 b	0.55 ± 0.00 d
Catechin	3.0	289.1, 203.1 109.1	0.05 ± 0.00 a	0.05 ± 0.00 a	0.05 ± 0.00 a	0.04 ± 0.00 b	0.05 ± 0.00 a	0.04 ± 0.00 b	0.05 ± 0.00 a
Gallic acid	3.7	169.0, 125.0	0.02 ± 0.00 b	0.03 ± 0.00 ab	0.03 ± 0.00 ab	0.03 ± 0.00 ab	0.03 ± 0.00 ab	0.03 ± 0.00 ab	0.04 ± 0.00 a
*p*-hydroxybenzoic acid	4.2	137.0, 108.0	0.01 ± 0.00 b	0.01 ± 0.00 b	0.01 ± 0.00 b	0.01 ± 0.00 b	0.01 ± 0.00 b	0.01 ± 0.00 b	0.02 ± 0.0 a
Genistein	5.2	269.0, 133.0	1.43 ± 0.01 c	1.20 ± 0.01 d	1.63 ± 0.00 b	1.37 ± 0.01 c	1.68 ± 0.01 a	1.42 ± 0.01 c	1.64 ± 0.01 b
Daidzein	5.6	255.1, 91.0	0.23 ± 0.00 cd	0.21 ± 0.00 d	0.31 ± 0.00 a	0.24 ± 0.00 c	0.30 ± 0.00 ab	0.28 ± 0.00 b	0.30 ± 0.00 ab
Matairesinol	6.2	357.1, 122.0 83.1	0.27 ± 0.00 d	0.23 ± 0.00 e	0.37 ± 0.00 ab	0.31 ± 0.00 c	0.35 ± 0.00 b	0.38 ± 0.00 a	0.32 ± 0.00 c
Methyl gallate	7.1	167.0, 140.0 124.0, 111.1	0.02 ± 0.00 b	0.02 ± 0.00 b	0.02 ± 0.00 b	0.02 ± 0.00 b	0.04 ± 0.00 a	0.05 ± 0.00 a	0.04 ± 0.00 a
Secoisolariciresinol	9.6	361.2, 165.0 346.1, 121.1	0.10 ± 0.00 ab	0.10 ± 0.00 ab	0.10 ± 0.00 ab	0.07 ± 0.00 c	0.10 ± 0.00 ab	0.11 ± 0.00 a	0.11 ± 0.00 a
Ethyl gallate	12.1	197.0, 124.0 169.0	0.02 ± 0.00 ab	0.02 ± 0.00 ab	0.03 ± 0.00 a	0.02 ± 0.00 ab	0.03 ± 0.00 a	0.02 ± 0.00 ab	0.02 ± 0.00 ab
Kaempferol	14.1	285.1, 151.0	0.02 ± 0.00 b	0.01 ± 0.00 b	0.02 ± 0.00 b	0.02 ± 0.00 b	0.04 ± 0.00 a	0.04 ± 0.00 a	0.03 ± 0.00 ab
Protocatechuic acid	14.5	315.1, 123.0 108.0	0.004 ± 0.000 b	0.004 ± 0.000 b	0.004 ± 0.00 b	0.004 ± 0.000 b	0.01 ± 0.00 a	0.001 ± 0.00 d	0.002 ± 0.000 c
Epicatechin gallate	14.7	441.1, 331.1 289.1, 168.9	0.04 ± 0.00 ab	0.03 ± 0.00 b	0.04 ± 0.00 ab	0.03 ± 0.00 b	0.05 ± 0.00 a	0.03 ± 0.00 b	0.04 ± 0.00 ab
Epigallocatechin gallate	16.0	457.1, 331.1 287.1, 169.1	0.12 ± 0.03 e	0.11 ± 0.00 e	0.17 ± 0.00 d	0.18 ± 0.00 cd	0.20 ± 0.00 b	0.33 ± 0.00 a	0.19 ± 0.00 bc
*p*-Coumaric acid	16.2	163.0, 146.0 119.1	0.04 ± 0.00 b	0.03 ± 0.00 b	0.04 ± 0.00 b	0.04 ± 0.00 b	0.06 ± 0.00 a	0.06 ± 0.00 a	0.06 ± 0.00 a
Rosmarinic acid	16.7	359.1, 197.1 179.1	LDL	LDL	LDL	LDL	LDL	0.002 ± 0.000	LDL
Quercetin	17.2	301.1, 179.1 151.1	0.06 ± 0.00 cd	0.05 ± 0.00 d	0.07 ± 0.00 c	0.06 ± 0.00 cd	0.09 ± 0.00 b	0.26 ± 0.00 a	0.10 ± 0.00 b
Formononetin	17.4	267.1, 252.1 191.1	1.52 ± 0.00 c	1.26 ± 0.01 d	1.31 ± 0.01 d	1.52 ± 0.01 c	2.13 ± 0.01 b	3.26 ± 0.01 a	2.06 ± 0.01 b
Biochanin A	18.9	283.1, 268.1 109.1	1.37 ± 0.01 d	1.07 ± 0.01 e	1.04 ± 0.00 e	1.48 ± 0.01 c	1.85 ± 0.01 b	2.44 ± 0.03 a	1.87 ± 0.02 b
Sinapic acid	21.3	223.1, 208.0	0.005 ± 0.000 e	0.005 ± 0.000 e	0.005 ± 0.000 e	0.04 ± 0.00 ab	0.02 ± 0.00 cd	0.03 ± 0.00 bc	0.05 ± 0.00 a
Eriocitrin	21.5	595.2, 287.1 151.0	0.005 ± 0.000 b	LDL	LDL	0.01 ± 0.00 a	0.01 ± 0.00 a	0.01 ± 0.00 a	0.01 ± 0.00 a
Ellagic acid	22.5	301.0, 229.0 185.0, 145.0	0.004 ± 0.000 b	0.004 ± 0.000 b	0.003 ± 0.000 c	0.01 ± 0.00 a	0.002 ± 0.000 d	0.003 ± 0.000 c	0.003 ± 0.000 c
Rutin	27.0	609.1, 301.1 151.1	0.20 ± 0.00 b	0.26 ± 0.00 a	0.27 ± 0.00 a	0.21 ± 0.00 b	0.27 ± 0.00 a	0.21 ± 0.00 b	0.27 ± 0.00 a
			San Antonio						
Chlorogenic acid	1.9	353.1, 191.1 179.0	0.33 ± 0.01 b	0.33 ± 0.00 b	0.33 ± 0.01 b	0.38 ± 0.00 a	0.25 ± 0.00 c	0.34 ± 0.01 b	0.40 ± 0.00 a
Epicatechin	2.0	289.1, 245.1 123.1	0.40 ± 0.00 b	0.40 ± 0.00 b	0.37 ± 0.00 c	0.40 ± 0.00 b	0.18 ± 0.00 d	0.41 ± 0.00 ab	0.45 ± 0.02 a
Catechin	3.0	289.1, 203.1 109.1	0.03 ± 0.00 a	0.03 ± 0.00 a	0.03 ± 0.00 a	0.03 ± 0.00 a	0.02 ± 0.00 a	0.03 ± 0.00 a	0.03 ± 0.00 a
Gallic acid	3.7	169.0, 125.0	0.01 ± 0.00 a	0.01 ± 0.00 a	0.01 ± 0.00 a	0.01 ± 0.00 a	0.01 ± 0.00 a	0.01 ± 0.00 a	0.01 ± 0.00 a
*p*-hydroxybenzoic acid	4.2	137.0, 108.0	0.01 ± 0.00 a	0.02 ± 0.00 a	0.02 ± 0.00 a	0.02 ± 0.00 a	0.02 ± 0.00 a	0.02 ± 0.00 a	0.02 ± 0.00 a
Genistein	5.2	269.1, 159.1 133.1	1.84 ± 0.01 d	2.03 ± 0.01 c	1.86 ± 0.00 d	2.42 ± 0.01 b	2.42 ± 0.01 b	2.02 ± 0.01 c	2.49 ± 0.02 a
Daidzein	5.6	253.1, 137.1	0.35 ± 0.00 c	0.37 ± 0.00 c	0.35 ± 0.00 c	0.46 ± 0.02 a	0.42 ± 0.00 b	0.40 ± 0.00 b	0.47 ± 0.01 a
Matairesinol	6.2	357.1, 122.0 83.1	0.27 ± 0.00 c	0.27 ± 0.00 c	0.27 ± 0.00 c	0.32 ± 0.00 b	0.38 ± 0.00 a	0.25 ± 0.00 c	0.39 ± 0.00 a
Methyl gallate	7.1	167.0, 140.0 124.0, 111.1	0.05 ± 0.00 a	0.04 ± 0.00 ab	0.03 ± 0.00 b	0.03 ± 0.00 b	0.05 ± 0.00 a	0.04 ± 0.00 ab	0.04 ± 0.00 ab
Secoisolariciresinol	9.6	361.2, 165.0 346.1, 121.1	0.11 ± 0.00 c	0.19 ± 0.00 a	0.14 ± 0.00 b	0.13 ± 0.00 b	0.13 ± 0.00 b	0.13 ± 0.00 b	0.11 ± 0.00 c
Ethyl gallate	12.1	197.0, 124.0 169.0	0.02 ± 0.00 b	0.04 ± 0.00 a	0.03 ± 0.00 ab	0.02 ± 0.00 b	0.02 ± 0.00 b	0.02 ± 0.00 b	0.04 ± 0.00 a
Kaempferol	14.1	285.1, 151.0	0.05 ± 0.00 bc	0.05 ± 0.00 bc	0.06 ± 0.00 b	0.04 ± 0.00 c	0.05 ± 0.00 bc	0.05 ± 0.00 bc	0.08 ± 0.00 a
Protocatechuic acid	14.5	315.1, 123.0 108.0	0.02 ± 0.00 a	0.02 ± 0.00 a	0.02 ± 0.00 a	0.02 ± 0.00 a	0.02 ± 0.00 a	0.02 ± 0.00 a	0.02 ± 0.00 a
Epicatechin gallate	14.7	441.1, 331.1 289.1, 168.9	0.06 ± 0.00 b	0.06 ± 0.00 b	0.09 ± 0.00 a	0.04 ± 0.00 c	0.05 ± 0.00 bc	0.06 ± 0.00 b	0.09 ± 0.00 a
Epigallocatechin gallate	16.0	457.1, 331.1 287.1, 169.1	0.15 ± 0.00 c	0.15 ± 0.00 c	0.16 ± 0.00 c	0.12 ± 0.00 d	0.25 ± 0.00 a	0.13 ± 0.00 d	0.19 ± 0.00 b
*p*-Coumaric acid	16.2	163.0, 146.0 119.1	0.06 ± 0.00 ab	0.06 ± 0.00 ab	0.06 ± 0.00 ab	0.03 ± 0.03 c	0.07 ± 0.00 a	0.05 ± 0.00 b	0.07 ± 0.00 a
Rosmarinic acid	16.7	359.1, 197.1 179.1	0.002 ± 0.000 a	0.002 ± 0.000 a	LDL	0.002 ± 0.000 a	0.002 ± 0.000 a	0.002 ± 0.000 a	0.001 ± 0.000 b
Quercetin	17.2	301.1, 179.1 151.1	0.14 ± 0.00 d	0.12 ± 0.00 e	0.26 ± 0.00 b	0.10 ± 0.00 f	0.17 ± 0.00 c	0.07 ± 0.00 g	0.32 ± 0.00 a
Formononetin	17.4	267.1, 252.1 191.1	2.53 ± 0.00 e	2.57 ± 0.01 d	3.00 ± 0.00 c	2.07 ± 0.00 f	3.39 ± 0.01 b	1.84 ± 0.02 g	3.66 ± 0.02 a
Biochanin A	18.9	283.1, 268.1 211.1, 109.1	1.96 ± 0.01 c	2.35 ± 0.01 b	1.77 ± 0.01 d	1.75 ± 0.02 d	2.96 ± 0.01 a	1.31 ± 0.02 e	2.39 ± 0.02 b
Sinapic acid	21.3	223.1, 208.0	0.03 ± 0.00 a	0.02 ± 0.00 ab	0.02 ± 0.00 ab	0.01 ± 0.00 bc	0.02 ± 0.00 ab	0.01 ± 0.00 bc	0.02 ± 0.00 ab
Eriocitrin	21.5	595.2, 287.1 151.1	0.01 ± 0.00 a	0.01 ± 0.00 a	0.005 ± 0.000 b	0.01 ± 0.00 a	0.003 ± 0.000 c	LDL	0.003 ± 0.000 c
Ellagic acid	22.5	301.0, 229.0 185.0, 145.0	0.004 ± 0.000 b	0.01 ± 0.00 a	0.01 ± 0.00 a	0.01 ± 0.00 a	0.01 ± 0.00 a	0.01 ± 0.00 a	0.01 ± 0.00 a
Rutin)	27.0	609.1, 301.1 151.1	0.28 ± 0.00 a	0.29 ± 0.00 a	0.29 ± 0.00 a	0.29 ± 0.00 a	0.29 ± 0.00 a	0.29 ± 0.00 a	0.29 ± 0.00 a

Data are presented as mean ± standard deviation of three experimental replicates. Results are expressed as mg/g dry flour. Different letters in the same row indicate significant difference (*p* < 0.05) by Tukey test. Control: germinated seeds with distilled water. SA: salicylic acid, CH: chitosan, H_2_O_2_: hydrogen peroxide. Samples were analyzed on fourth day of germination. LDL: lower than detection limit.

**Table 4:** Phytosterol profile of chemically stressed chickpea sprouts (CS) of three Mexican cultivars.

Phytosterols	Retention Time	*m*/*z*	Control	1 mM SA	2 mM SA	20 mM H_2_O_2_	30 mM H_2_O_2_	3.3 μM CH	7 μM CH
			Blanoro						
Brassicasterol	2.1	381.4, 297.3 147.3, 84.3	16.38 ± 0.45 d	37.46 ± 0.39 a	25.37 ± 0.14 c	36.67 ± 0.69 ab	36.05 ± 0.84 b	36.87 ± 0.75 ab	36.61 ± 0.79 ab
Ergosterol	2.3	379.4, 295.3 184.3, 158.4	4.26 ± 0.16 e	23.72 ± 0.15 a	19.76 ± 0.42 c	22.41 ± 0.32 b	23.05 ± 0.70 ab	20.59 ± 0.29 c	17.90 ± 0.20 d
Fucosterol	2.7	395.4, 355.3 303.3, 195.3 121.3	2.65 ± 0.06 c	2.55 ± 0.03 c	6.62 ± 0.15 b	12.10 ± 0.10 a	2.21 ± 0.07 d	1.65 ± 0.07 e	1.61 ± 0.07 e
Avenasterol	3.8	395.4, 109.3 81.3	40.10 ± 0.93 c	48.38 ± 0.27 b	61.80 ± 0.46 a	49.88 ± 1.73 b	35.83 ± 0.24 d	19.38 ± 0.55 e	19.57 ± 0.29 e
Stigmasterol	6.3	395.3, 91.3 83.3	2.14 ± 0.15 e	3.67 ± 0.05 b	3.21 ± 0.09 c	2.04 ± 0.04 e	2.52 ± 0.09 d	3.62 ± 0.03 b	5.34 ± 0.22 a
β-Sitosterol	7.3	397.3, 95.3 91.3	165.63 ± 0.08 c	198.86 ± 0.58 a	199.74 ± 0.98 a	195.40 ± 0.65 b	197.04 ± 0.99 ab	197.95 ± 0.55 a	199.74 ± 0.22 a
β-Campesterol	87	383.3, 91.3 81.3	17.97 ± 0.07 e	30.28 ± 0.40 b	27.90 ± 0.27 c	20.97 ± 0.62 d	20.44 ± 0.06 d	29.54 ± 0.65 bc	35.95 ± 0.36 a
Avenasterol glucopyranoside	16.9	395.4, 109.3 81.3	35.88 ± 0.14 a	22.49 ± 0.45 d	20.52 ± 0.29 e	25.61 ± 0.36 c	34.67 ± 0.28 b	26.55 ± 0.91 c	35.46 ± 0.22 a
Stigmastanol (sitostanol)	17.1	399.4, 397.3 149.1, 95.3 91.3	4.93 ± 0.19 b	3.65 ± 0.14 d	4.53 ± 0.01 c	4.91 ± 0.17 b	6.65 ± 0.08 a	5.11 ± 0.09 b	5.09 ± 0.03 b
Sitosteryl glucopyranoside	19.7	594.4, 397.3, 95.4	15.14 ± 0.20 a	9.42 ± 0.21 d	8.50 ± 0.12 e	11.49 ± 0.26 b	10.65 ± 0.19 c	6.95 ± 0.17 f	8.73 ± 0.08 e
Campesteryl glucopyranoside	20.1	580.4, 383.3, 91.3	20.62 ± 0.62 e	17.42 ± 0.17 f	17.44 ± 0.17 f	25.43 ± 0.31 d	30.68 ± 0.64 c	32.93 ± 0.32 b	50.43 ± 1.77 a
Stigmasteryl glucopyranoside	25.6	592.4, 395.3 83.3	LDL	LDL	3.35 ± 0.07 b	0.79 ± 0.02 d	3.37 ± 0.05 b	1.04 ± 0.05 c	4.71 ± 0.19 a
			Patron						
Brassicasterol	2.1	381.4, 297.3 147.3, 84.3	14.10 ± 0.20 e	20.78 ± 1.11 d	6.88 ± 0.32 g	45.09 ± 0.17 b	75.91 ± 1.09 a	25.67 ± 0.34 c	9.86 ± 0.15 f
Ergosterol	2.3	379.4, 295.3 184.3, 158.4	LDL	LDL	LDL	12.79 ± 0.08 c	34.04 ± 0.45 a	19.33 ± 0.05 b	LDL
Fucosterol	2.7	395.4, 355.3 303.3, 195.3 121.3	1.05 ± 0.04 g	10.10 ± 0.20 f	33.46 ± 0.38 b	34.84 ± 0.54 a	14.92 ± 0.03 d	26.36 ± 1.33 c	12.99 ± 0.04 e
Avenasterol	3.8	395.4, 109.3 81.3	2.16 ± 0.01 d	LDL	1.81 ± 0.11 f	3.01 ± 0.14 b	3.72 ± 0.12 a	2.67 ± 0.03 c	2.03 ± 0.02 e
Stigmasterol	6.3	395.3, 91.3 83.3	2.04 ± 0.02 d	29.14 ± 0.84 b	31.00 ± 0.13 a	25.57 ± 0.25 c	29.71 ± 0.22 b	24.43 ± 0.94 c	24.73 ± 0.32 c
β-Sitosterol	7.3	397.3, 95.3 91.3	198.34 ± 0.36 e	231.89 ± 3.18 c	227.17 ± 0.58 d	226.44 ± 0.92 d	246.79 ± 2.64 b	293.15 ± 1.81 a	296.48 ± 1.52 a
β-Campesterol	87	383.3, 91.3 81.3	60.57 ± 0.41 b c	56.90 ± 0.32 d	59.04 ± 1.04 c	61.92 ± 0.74 b	56.33 ± 0.30 de	55.97 ± 0.46 e	65.62 ± 0.81 a
Avenasterol glucopyranoside	16.9	395.4, 109.3 81.3	27.93 ± 0.16 e	29.27 ± 0.11 d	37.16 ± 0.24 a	35.85 ± 0.37 b	30.56 ± 0.20 c	23.08 ± 0.21 f	26.72 ± 0.40 e
Stigmastanol (sitostanol)	17.1	399.4, 397.3 149.1, 95.3 91.3	3.00 ± 0.07 d	2.18 ± 0.17 e	4.09 ± 0.04 b	4.10 ± 0.05 b	3.47 ± 0.10 c	4.86 ± 0.20 a	3.91 ± 0.14 b
Sitosteryl glucopyranoside	19.7	594.4, 397.3, 95.4	54.65 ± 0.16 a	10.51 ± 0.17 f	26.06 ± 0.94 c	22.16 ± 0.44 d	24.98 ± 0.35 c	16.35 ± 0.06 e	30.06 ± 0.23 b
Campesteryl glucopyranoside	20.1	580.4, 383.3, 91.3	133.77 ± 2.45 a	10.28 ± 0.19 e	83.37 ± 0.76 c	72.98 ± 0.52 d	73.73 ± 0.93 d	9.92 ± 0.24 e	91.53 ± 0.88 b
Stigmasteryl glucopyranoside	25.6	592.4, 395.3 83.3	3.24 ± 0.06 b	11.71 ± 0.23 a	11.80 ± 0.87 a	3.49 ± 0.11 b	LDL	2.91 ± 0.03 c	2.87 ± 0.07 c
			San Antonio						
Brassicasterol	2.1	381.4, 297.3 147.3, 84.3	24.30 ± 0.21 a	23.75 ± 0.03 a	16.82 ± 0.53 c	9.24 ± 0.16 d	18.54 ± 0.35 b	23.25 ± 0.79 a	16.29 ± 0.49 c
Ergosterol	2.3	379.4, 295.3 184.3, 158.4	8.35 ± 0.21 e	4.92 ± 0.05 f	33.16 ± 0.60 c	46.33 ± 0.65 b	34.28 ± 0.79 c	51.69 ± 0.90 a	29.95 ± 0.66 d
Fucosterol	2.7	395.4, 355.3 303.3, 195.3 121.3	40.16 ± 0.57 e	93.50 ± 1.21 a	73.68 ± 0.5 c	88.72 ± 1.01 b	75.80 ± 1.07 c	93.76 ± 1.01 a	59.14 ± 0.29 d
Avenasterol	3.8	395.4, 109.3 81.3	2.60 ± 0.02 e	6.21 ± 0.10 b	6.72 ± 0.17 a	3.50 ± 0.15 d	6.54 ± 0.09 a	1.50 ± 0.13 f	5.04 ± 0.06 c
Stigmasterol	6.3	395.3, 91.3 83.3	7.00 ± 0.04 b	3.99 ± 0.17 c	3.76 ± 0.05 c	3.71 ± 0.10 cd	3.55 ± 0.09 de	10.38 ± 0.15 a	3.37 ± 0.16 e
β-Sitosterol	7.3	397.3, 95.3 91.3	149.73 ± 1.87 d	197.94 ± 3.74 b	150.13 ± 2.51 d	196.91 ± 3.74 b	166.38 ± 2.31 c	203.71 ± 2.03 b	255.85 ± 1.80 a
β-Campesterol	87	383.3, 91.3 81.3	25.72 ± 0.38 d	30.21 ± 0.43 b	26.10 ± 0.75 d	31.35 ± 0.37 b	27.36 ± 0.20 cd	38.58 ± 0.10 a	24.86 ± 0.70 d
Avenasterol glucopyranoside	16.9	395.4, 109.3 81.3	36.04 ± 0.67 bc	51.70 ± 1.18 a	30.21 ± 0.27 d	48.96 ± 2.10 a	35.21 ± 0.34 c	37.72 ± 0.39 b	21.02 ± 0.86 e
Stigmastanol (sitostanol)	17.1	399.4, 397.3 149.1, 95.3 91.3	6.20 ± 0.13 d	8.27 ± 0.15 a	6.44 ± 0.13 cd	8.20 ± 0.15 a	6.64 ± 0.09 bc	6.92 ± 0.20 b	3.52 ± 0.03 e
Sitosteryl glucopyranoside	19.7	594.4, 397.3, 95.4	37.14 ± 0.40 c	30.66 ± 0.24 d	38.52 ± 0.49 b	27.69 ± 0.51 e	24.69 ± 0.29 f	45.57 ± 0.36 a	30.86 ± 0.85 d
Campesteryl glucopyranoside	20.1	580.4, 383.3, 91.3	139.09 ± 1.39 c	140.69 ± 1.26 c	146.37 ± 1.50 b	136.15 ± 0.72 d	114.73 ± 2.01 e	151.01 ± 1.55 a	99.98 ± 4.30 f
Stigmasteryl glucopyranoside	25.6	592.4, 395.3 83.3	4.33 ± 0.03 d	1.97 ± 0.02 f	4.33 ± 0.01 d	6.79 ± 0.11 b	5.74 ± 0.09 c	8.64 ± 0.14 a	2.39 ± 0.03 e

Data are presented as mean ± standard deviation of three experimental replicates. Results are expressed as μg/g dry flour. Different letters in the same row indicate significant difference by (*p* < 0.05) Tukey test. Control: germinated seeds with distilled water. SA: salicylic acid, CH: chitosan, H_2_O_2_: hydrogen peroxide. Samples were analyzed on fourth day of germination. LDL: lower than detection limit.

**Table 5:** Saponin profile of chemically stressed chickpea sprouts (CS) of three Mexican cultivars.

Saponins	Retention Time	*m*/*z*	Control	1 mM SA	2 mM SA	20 mM H_2_O_2_	30 mM H_2_O_2_	3.3 μM CH	7 μM CH
			Blanoro						
Phaseoside I	9.8	1252.5 1091.5, 959.5	134.42 ± 3.12 d	177.14 ± 0.95 b	217.70 ± 2.33 a	158.87 ± 2.30 c	120.67 ± 1.99 e	131.73 ± 1.91 d	124.93 ± 3.20 e
Soyasaponin Bb (I)	11.7	941.5, 489.5	142.71 ± 0.70 a	124.51 ± 2.38 b	115.78 ± 0.78 c	113.04 ± 0.78 cd	124.14 ± 1.21 b	107.57 ± 3.51 d	93.06 ± 1.78 e
Soyasaponin Ba (V)	13.1	957.5, 795.5 633.5	253.45 ± 1.65 c	285.74 ± 13.27 a	241.45 ± 5.37 d	263.83 ± 3.68 b	272.78 ± 7.80 ab	233.52 ± 2.92 e	237.45 ± 2.43 de
Soyasaponin Af	13.7	1273.6, 943.5 811.5, 329.5	686.75 ± 7.89 d	816.64 ± 11.57 b	948.68 ± 7.12 a	766.53 ± 11.45 c	944.52 ± 11.68 a	507.23 ± 2.84 e	473.94 ± 4.21 f
Soyasaponin Bd	14.9	955.5, 793.5 631.5	61.70 ± 0.21 d	74.08 ± 0.98 b	49.52 ± 1.03 f	63.47 ± 0.41 c	48.76 ± 1.47 f	75.84 ± 0.26 a	56.06 ± 0.93 e
Soyasaponin βg (VI)	19.2	1067.5, 533.5	771.86 ± 4.91 c	835.41 ± 6.75 c	961.30 ± 7.25 a	913.43 ± 20.92 b	854.98 ± 18.14 c	621.34 ± 8.72 d	542.97 ± 4.85 e
Soyasaponin αg	27.2	1083.5, 561.5 559.5, 541.5	58.40 ± 0.18 f	80.90 ± 1.49 e	87.18 ± 1.12 d	99.28 ± 0.72 c	48.72 ± 1.14 g	121.05 ± 0.65 b	147.00 ± 4.02 a
			Patron						
Phaseoside I	9.8	1252.5 1091.5, 959.5	22.37 ± 0.51 e	45.20 ± 0.97 d	52.95 ± 1.81 c	42.46 ± 0.96 d	131.97 ± 3.66 b	168.89 ± 3.93 a	45.30 ± 1.39 d
Soyasaponin Bb (I)	11.7	941.5, 489.5	59.92 ± 1.66 cd	88.31 ± 2.96 a	62.29 ± 0.97 bc	65.18 ± 1.36 b	64.90 ± 1.18 b	92.33 ± 1.98 a	56.81 ± 2.00 d
Soyasaponin Ba (V)	13.1	957.5, 795.5 633.5	178.81 ± 3.71 b	139.55 ± 6.36 d	147.19 ± 0.46 c	144.88 ± 2.96 cd	172.60 ± 3.27 b	185.67 ± 3.00 a	148.02 ± 1.19 c
Soyasaponin Af	13.7	1273.6, 943.5 811.5, 329.5	269.79 ± 4.37 d	277.66 ± 3.27 d	298.36 ± 6.92 c	298.80 ± 2.96 c	354.00 ± 2.54 b	411.43 ± 5.00 a	347.52 ± 9.03 b
Soyasaponin Bd	14.9	955.5, 793.5 631.5	102.48 ± 1.18 d	117.30 ± 0.50 c	119.53 ± 1.63 c	120.36 ± 2.69 c	102.26 ± 1.16 d	260.03 ± 1.84 a	249.29 ± 4.92 b
Soyasaponin βg (VI)	19.2	1067.5, 533.5	306.43 ± 6.02 c	326.42 ± 4.84 b	302.33 ± 6.63 cd	255.05 ± 4.69 e	333.63 ± 5.36 b	384.49 ± 4.39 a	290.92 ± 5.64 d
Soyasaponin αg	27.2	1083.5, 561.5 559.5, 541.5	249.20 ± 2.53 d	261.45 ± 3.21 d	230.39 ± 3.33 e	318.39 ± 8.29 b	383.43 ± 6.23 a	295.63 ± 6.68 c	310.84 ± 4.19 b
			San Antonio						
Phaseoside I	9.8	1252.5 1091.5, 959.5	65.78 ± 3.95 f	93.03 ± 3.58 e	109.19 ± 2.54 d	122.39 ± 3.23 c	114.12 ± 2.71 d	147.15 ± 1.46 b	170.99 ± 1.01 a
Soyasaponin Bb (I)	11.7	941.5, 489.5	89.10 ± 3.65 c	109.13 ± 2.36 b	153.79 ± 5.69 a	109.54 ± 3.35 b	89.58 ± 3.61 c	79.98 ± 0.63 d	67.06 ± 1.18 e
Soyasaponin Ba (V)	13.1	957.5, 795.5 633.5	231.78 ± 5.94 bc	250.51 ± 3.33 a	205.03 ± 4.02 e	215.71 ± 5.33 d	225.54 ± 4.30 cd	237.32 ± 0.81 b	234.94 ± 2.68 b
Soyasaponin Af	13.7	1273.6, 943.5 811.5, 329.5	473.44 ± 7.25 g	719.01 ± 5.82 b	613.75 ± 9.27 d	530.90 ± 2.19 f	571.67 ± 9.85 e	678.25 ± 11.80 c	752.27 ± 8.29 a
Soyasaponin Bd	14.9	955.5, 793.5 631.5	39.65 ± 0.90 b	36.74 ± 0.49 c	LDL	37.52 ± 1.01 bc	LDL	47.82 ± 1.39 a	47.36 ± 1.15 a
Soyasaponin βg (VI)	19.2	1067.5, 533.5	506.50 ± 7.29 e	638.01 ± 8.65 b	520.03 ± 4.35 e	602.69 ± 5.72 c	606.66 ± 5.57 c	566.98 ± 10.66 d	685.96 ± 3.70 a
Soyasaponin αg	27.2	1083.5, 561.5 559.5, 541.5	127.23 ± 3.76 cd	138.39 ± 4.31 b	176.72 ± 3.30 a	132.01 ± 1.23 c	86.96 ± 3.38 e	91.62 ± 7.30 e	125.29 ± 3.54 d

Data are presented as mean ± standard deviation of three experimental replicates. Results are expressed as μg/g dry flour. Different letters in the same row indicate significant difference by (*p* < 0.05) Tukey test. Control: germinated seeds with distilled water. SA: salicylic acid, CH: chitosan, H_2_O_2_: hydrogen peroxide. Samples were analyzed on fourth day of germination. LDL: lower than detection limit.

## Data Availability

The data that support the findings of this study are available from the corresponding author, [M.R.-G.], upon reasonable request.
